# Quality of Life in Hidradenitis Suppurativa: An Update

**DOI:** 10.3390/ijerph18116131

**Published:** 2021-06-06

**Authors:** Pavel V. Chernyshov, Andrew Y. Finlay, Lucia Tomas-Aragones, Francoise Poot, Francesca Sampogna, Servando E. Marron, Sergey V. Zemskov, Damiano Abeni, Thrasyvoulos Tzellos, Jacek C. Szepietowski, Christos C. Zouboulis

**Affiliations:** 1 Department of Dermatology and Venereology, Bogomolets National Medical University, 01601 Kiev, Ukraine; 2 Division of Infection and Immunity, School of Medicine, Cardiff University, Cardiff CF14 4XN, UK; finlayay@cardiff.ac.uk; 3 Department of Psychology, University of Zaragoza, 50009 Zaragoza, Spain; luciatomas@cop.es; 4 Department of Dermatology, University Hospital Erasme, 1070 Brussels, Belgium; psychodermato@hotmail.com; 5 Clinical Epidemiology Unit, IDI-IRCCS, 00167 Rome, Italy; fg.sampogna@gmail.com (F.S.); d.abeni@idi.it (D.A.); 6 Aragon Psychodermatology Research Group (GAI + PD), Department of Dermatology, University Hospital Miguel Servet, 50009 Zaragoza, Spain; semarron@aedv.es; 7 Department of General Surgery, Bogomolets National Medical University, 01601 Kiev, Ukraine; s.zemskov@nmu.ua; 8 Department of Dermatology, NLSH University Hospital, 8092 Bodø, Norway; thrasyvoulos.tzellos@nordlandssykehuset.no; 9 European Hidradenitis Suppurativa Foundation e.V., 06847 Dessau, Germany; jacek.szepietowski@umed.wroc.pl (J.C.S.); christos.zouboulis@mhb-fontane.de (C.C.Z.); 10 Department of Dermatology, Venereology and Allergology, Wroclaw Medical University, 50-368 Wroclaw, Poland; 11 Departments of Dermatology, Venereology, Allergology and Immunology, Dessau Medical Center, Brandenburg Medical School Theodor Fontane and Faculty of Health Sciences, 06847 Dessau, Germany

**Keywords:** hidradenitis suppurativa, quality of life, impact, treatment, biologics, surgery

## Abstract

Knowledge on hidradenitis suppurativa/acne inversa (HS) is rapidly increasing. HS has a profound impact on patients and their family life. Several factors, such as comorbidities, unemployment and HS severity, make this impact even more severe. The most widely used instrument to measure this impact is the dermatology-specific DLQI. We also identified six HS-specific health-related quality of life (HRQoL) instruments. Of them, HIDRAdisk, HSIA, HiSQOL and HSQoL-24 are better validated but there is still lack of experience of its use. Several treatment methods showed positive effect on patients’ HRQoL. Surgery remains a method with a substantial positive effect on HRQoL. Several studies confirming a positive effect of adalimumab on the HRQoL of patients with HS were published during the last three years. Data on the influence of several other biologics on HRQoL of HS patients are controversial or based on studies with a small number of patients.

## 1. Introduction

**Figure 1:**
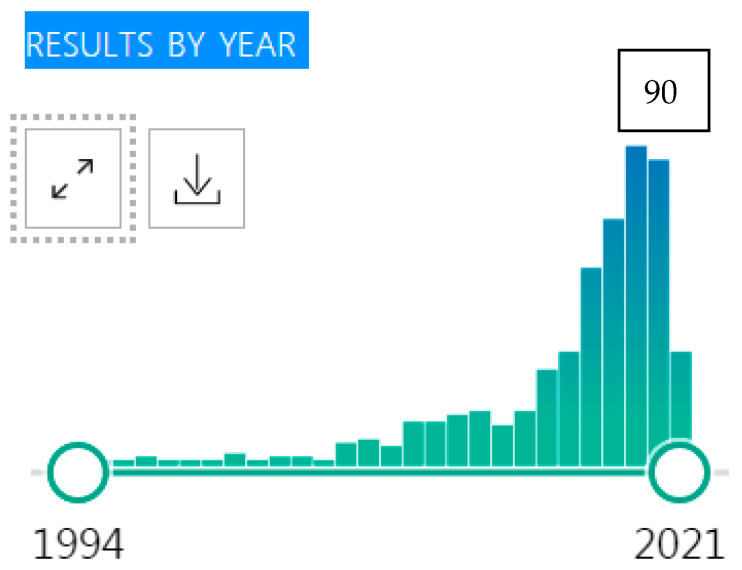
Number of publications on quality of life (QoL) in hidradenitis suppurativa (HS) per year in PubMed (https://pubmed.ncbi.nlm.nih.gov/?term=hidradenitis%20suppurativa%2C%20quality%20of%20life; accessed on 2 April 2021).

Knowledge about hidradenitis suppurativa/acne inversa (HS) is rapidly increasing. Following the widely accepted Dessauer definition and the establishment of simple but highly accurate diagnostic criteria for earlier recognition of the disease [1], a resolution of the contradictory epidemiological data has been achieved [2] and a practical framework to optimise therapy has been proposed [3]. Highly sophisticated molecular studies have achieved a better understanding of HS pathophysiology [4], including the identification of relevant molecular expression patterns, which may allow the possibility of bringing personalised medicine to HS management [5]. There are novel validated criteria for severity classification, such as the International Hidradenitis Suppurativa Severity Score System (IHS4) [6], and robust clinical outcome measures that allow better monitoring of the disease course during clinical studies. Future treatment target molecules have been proposed [7], and experimental human models for in vitro/ex vivo studies of HS are under development [8]. Visual technology, such as standardised photography, ultrasound and thermography [9,10,11,12], together with digital technology for patient outcome measures, such as daily digital assessment of pain [13], have the potential to markedly improve the objective evaluation of HS severity assessment. On the other hand, HS continues to be the skin disease that causes the greatest impairment of quality of life (QoL) [14] and methods to measure this impairment, although developing, have not yet improved to the extent of other physical aspects of the disease [15,16]. It is likely that many studies on the QoL impact of HS will continue to be carried out: their rate of publication increased from 0–1 per year in the 1990s to 90 in 2019 (PubMed; Figure 1).

The European Academy of Dermatology and Venereology (EADV) Task Forces (TFs) on QoL and Patient Oriented Outcomes and Acne, Rosacea and HS concluded in their mutual position statement on QoL measurement in HS [15] that HS results in significant quality of life impairment (quimp), which is greater than in most other chronic skin diseases. Pain and other HS symptoms have the greatest influence on health-related (HR) QoL. Surgical treatment apparently has a substantial positive effect on the HRQoL of HS patients, when assessed over the long term. There is a lack of placebo-controlled clinical trials with sufficient participant numbers that assess different treatment methods and a lack of high-quality clinical trials in HS patients where HRQoL instruments have been used as outcome measures. The EADV TFs encouraged the further development, validation and use of other HS-specific, dermatology-specific and generic instruments, but such use should be based on the principles presented by the EADV TF on QoL and Patient Oriented Outcomes [15,17,18,19,20,21,22,23,24,25,26,27]. The aim of the present review is to present an update on recent QoL studies in HS.

## 2. Materials and Methods

Methods for identifying relevant publications were similar to those used previously in the preparation of the position statement of the EADV TFs on QoL and Patient Oriented Outcomes and Acne, Rosacea and HS on the QoL measurement in HS [15]. A literature search was performed using the PubMed database, which was searched from 2019 to March 2021 using the key word combinations: “hidradenitis suppurativa, quality of life” and “acne inversa, quality of life”. All publications written in English or those having English abstracts were considered.

Inclusion criteria:

- Articles with assessment of HRQoL in HS patients;
- Studies on the development and validation of HS-specific HRQoL instruments;
- Studies where HRQoL was studied in HS and other diseases and results on HS were presented and/or discussed separately.

Exclusion criteria:

- Review articles, guidelines and protocols;
- Studies without HRQoL assessment;
- Measurement of HRQoL in conditions other than HS;
- Studies where HRQoL was studied in HS and other diseases but results on HS were not presented and/or discussed separately.

All articles were primarily reviewed by the corresponding author. For those articles that fulfilled the inclusion and exclusion criteria, the study aim and design, the HRQoL instruments used, treatment methods (where appropriate) and the main study results related to HRQoL were recorded. The EADV TF on QoL and Patient Oriented Outcomes recommends using the word “quimp” [28] (quality of life impairment) in routine clinical work and research [29] and this word is also used in this article.

## 3. Results

### 3.1. HS-Specific HRQoL Instruments

We identified six HS-specific HRQoL instruments. The name, number of items and scales, recall period, scoring system and data on validation for each instrument are presented in Table 1.

A single item question, the ‘global item for assessing impact on quality of life of patients with hidradenitis suppurativa′ was proposed by Kirby et al. [36] and showed good convergent validity. The item was based on a 7-day recall period and the item responses based on a Likert scale (0–4). An unnamed non-validated 10-item questionnaire that focused on perianal problems (6 of 10 items) was adapted from an instrument used for Crohn′s disease and was used in a single study [37].

### 3.2. Treatment Effects on HRQoL of Patients with HS

#### 3.2.1. Biolologics and Immunomodulators

(a) Apremilast: There was no statistical difference in QoL measured by the DLQI between treatment and placebo groups after 16 weeks of apremilast treatment (mean difference, −3.4; 95% confidence interval, −9.0 to 2.3; *p* = 0.23) [38]. In contrast, an open-label study showed a significant improvement of DLQI scores after 24 weeks of apremilast treatment (11.6 to 5.4; *p* < 0.01) [39].

(b) Adalimumab: A cross-sectional study in three HS centres showed improvements (*p* = 0.0001) in the DLQI scores of those patients who had been taking adalimumab for at least six months [40]. In another study, the mean DLQI scores of HS patients treated with adalimumab decreased from 11.9 at week 0 to 6.6 at week 96 [41]. In a study carried out across 21 Italian centres, in which 389 patients with HS were treated with adalimumab, there were significant reductions in DLQI scores between baseline and weeks 16 and 52 (*p* < 0.0001 for both) [42]. The 24-week interim analysis from a phase 3 open-label trial of adalimumab in Japanese patients with moderate to severe HS showed HRQoL improvement: the mean DLQI at baseline was 5.5, and the mean (SD) score change from baseline was −2.2 (3.4) at 4 weeks and −0.5 (5.3) at 24 weeks [43]. However, it should be noted that the minimal clinically important difference (MCID) for the DLQI is a score change of 4 points [44]. An increase of adalimumab dosage from 40 mg/week to 80 mg/week subcutaneously led to HRQoL improvement as measured by the DLQI [45]. In a study comparing the effect of adalimumab versus rifampicin plus clindamycin on the HRQoL of HS patients, both groups achieved significant HRQoL improvement as measured by the Hidradisk [46]. In those subjects treated with adalimumab, the HIDRAdisk scores showed significantly better improvement in males compared to females (*p* < 0.001) [47].

(c) Ustekinumab: In 71.4% of patients DLQI scores improved on ustekinumab treatment [48].

(d) Secukinumab: An open-label pilot trial of secukinumab 300 mg administered subcutaneously weekly for 5 weeks, then every 4 weeks for 24 weeks was conducted in nine patients with moderate-to-severe HS. The median DLQI score decreased from 13 to 7 [49].

#### 3.2.2. Botulinum Toxin

Botulinum toxin type B was compared with placebo in the treatment of HS [50]. The DLQI improved from a median of 17 at baseline to 8 at 3 months in the botulinum toxin group, compared with an improvement from 13.5 to 11 in the placebo group (*p* < 0.05).

#### 3.2.3. Antibiotic and Corticosteroid

A study of the effect of two intralesional ultrasound-guided injections of triamcinolone plus lincomycin into areas affected by HS showed significant improvement of the SF-36 Bodily Pain scale from 36.2 at baseline to 53.9 at 4-week follow-up (*p* < 0.001) [51].

#### 3.2.4. Surgery

Data concerning QoL issues after surgery for HS are limited. The largest study amongst those recently published assessed 149 patients with Hurley stages I–III [52], but other recent studies involved much lower numbers of participants, with a maximum of 47 [53]. CO_2_ laser (a tissue sparing technique) was the most common treatment modality described in the study by Grimstad et al. [52]. The DLQI MCID of at least a four-point change was achieved in 55% of the patients. A single centre prospective study, conducted on 40 patients who underwent complete resection of the affected tissue by wide excision, leaving the wound for healing by secondary intention, demonstrated that the mean DLQI score did not significantly improve during the six-month follow-up period [54]. Another small study used the HSBOD scale in 19 patients to assess the impact of surgical intervention [55]. HSBOD scores were significantly higher in the symptoms and feelings domain in patients who had complex closure compared to those who had either secondary intention healing or who had split-thickness skin grafting. QoL after complex wound closure was measured in 27 patients who underwent pedicled perforator flap reconstruction after wide local excision (WLE). The mean DLQI score significantly improved after six months, from 21.3 ± 4.8 to 5.0 ± 3.0 at the last follow-up (*p* < 0.0001). Furthermore, DLQI scores were not influenced by complications such as having to have a further operation [56]. Another study compared QoL in 47 HS patients after WLE with either artificial dermal or pedicled perforator flap closure [53]. There was a significant increase in postoperative QoL, measured using the SF-36, in both groups (*p* < 0.05), but with greater QoL improvement in the perforator flap group (*p* < 0.001). The DLQI scores also revealed an improvement in QoL in both groups with greater improvement in the perforator flap group (*p* < 0.05) [53].

#### 3.2.5. Comparison of Different Treatment Methods

In 55% of HS patients treated surgically, a DLQI score change equivalent to the MCID (score change of 4) was achieved. In patients treated medically, 28% achieved the DLQI MCID and in patients receiving a combination of surgical and medical treatments, 48% achieved the DLQI MCID [52]. In the study on the effect of antibiotics and adalimumab on the HRQoL of HS patients, both groups achieved significant HRQoL improvement measured by the Hidradisk [46]. The three-arm parallel-group design trial showed that after 12 weeks, the decrease (improvement) in the mean DLQI score was significantly greater in the intense pulsed light + radiofrequency and radiofrequency alone groups compared to the intense pulsed light group [57]. HRQoL, assessed by the DLQI, improved more in patients treated with clindamycin than in the patients treated with clindamycin plus rifampicin [58]. ‘More marked’ HRQoL improvement, as measured by the DLQI, occurred in patients treated with tetracycline alone compared to patients treated with a combination of clindamycin and rifampicin [59].

### 3.3. QoL in Cohabitants of Patients with HS

QoL in cohabitants of patients with HS was independently studied in four studies from Poland, Italy, Spain and USA, using the Family Dermatology Life Quality Index (FDLQI) [60,61,62,63]. In the Polish study, the mean FDLQI for patients′ partners was 8.7 ± 6.8, indicating a moderate effect of HS on their lives. The analysis also revealed a positive correlation between FDLQI scores and the severity of HS, as measured by the Hurley Staging System. There was a strong positive correlation between FDLQI scores and disease severity as measured by the HSSI [60]. The mean FDLQI score of the cohabitant group in the Italian study was 10.1 (range 0–19). The FDLQI score of the cohabitants correlated with the DLQI score of patients (*r* = 0.88; *p* < 0.01) and was also significantly related to the Hurley stage (*r* = 0.69; *p* < 0.05). FDLQI scores were lower (i.e., less impact of QoL) in cohabitants with higher professional/university education (7.28 vs. 11.16; *p* = 0.037) than in those with lower educational attainment. The mean FDLQI scores were higher in partners (10.8) or spouses (8.1, *p* < 0.01) compared with parents [61]. In the Spanish study, the mean DLQI score of HS patients was 13.9 ± 9.5 and the mean FDLQI score was 10.5 ± 7.8. There was a significant association between DLQI and FDLQI scores [62]. The mean FDLQI scores for parents of children with HS in the US study was 9.9 (range 0–30). Caregivers’ lives were most impacted by the time spent caring for their child with HS and the personal emotional distress related to their child′s condition [63].

### 3.4. HS Impact on Work and Employment Status

In a study involving the international HS registry UNITE, only 60% of adults with HS were employed and of those, 64% reported at least some degree of impairment while working because of HS [64]. HS patients who were employed had better HRQoL as measured by the DLQI (mean score 11.0) than the unemployed (14.6, adjusted *p* = 0.003) [65]. Moderate to strong correlations were observed between reduction in QoL scores and the scores of the Work Productivity and Activity Impairment (WPAI) questionnaire modified for HS outcomes: presenteeism, overall work impairment and activity impairment (*r* = 0.50–0.77). The mean rank of activity impairment was 34.8 among patients with Hurley stage I, 60.1 for Hurley stage II and 64.0 for Hurley stage III, *p* < 0.0001. Unemployed patients had higher activity impairment compared with employed patients (mean rank: 61.0 vs. 42.6, *p* = 0.001) [66]. In another study, the DLQI, EuroQol-5 Dimensions scores and pain were significantly associated with presenteeism and at-work productivity loss [67]. Results from the Global Survey of Impact and Healthcare Needs (VOICE) project showed that 14.5% of HS patients were disabled due to their disease [68]. HS symptom severity strongly correlated with negative impact on work or schooling [69].

### 3.5. Correlation of QoL and Disease Severity in HS Patients

Jørgensen et al. [65] demonstrated that all 10 individual DLQI item scores increased significantly with increasing Hurley stage. Hurley stage III patients had significantly higher mean DLQI scores (20.2) compared to patients with Hurley stage I (11.3, *p* = 0.001) and II (13.9, *p* = 0.001) [70]. In another study, the only statistically significant difference in DLQI scores by Hurley stage was between stages 0 and 3 (*p* < 0.05) [71]. DLQI and physician-assessed International HS Severity Score System (IHS4) scores increased with increasing severity as assessed by the refined (seven-stage) Hurley classification. There was a significant positive correlation between both DLQI and IHS4 scores and with increasing severity assessed by the refined Hurley substages [72].

### 3.6. HRQoL in Different Age Groups of HS Patients

Young patients with HS reported a physical and mental health status similar to that of elderly people from the general population. Even patients with HS of mild-to-moderate clinical severity had a consistently worse health status than the reference population [72]. HS had a large effect (mean DLQI = 12.6) on the lives of adults and a moderate effect (mean CDLQI = 6.9) on the lives of adolescents. Approximately 58% of adults and 41% of adolescents had anxiety scores above the normal range. More than 45% of adult patients reported a significant emotional impact of HS that adversely affected their intimate relationships [64]. The average score of the Skindex-teen in children with HS was 45.7 (0–84). The greatest negative impacts on QoL were related to the appearance of the involved skin and to patients feeling frustrated by their HS [63].

### 3.7. Other Findings Related to QoL Assessment in HS Patients

Impaired QoL and reduced psychological well-being in HS patients were more strongly associated with the person′s beliefs about their illness than clinicians′ severity assessments [73]. Both pruritus and malodour in HS patients negatively correlated with HRQoL [74]. Higher stigmatisation scores correlated with worse HRQoL (r: 0.52, *p* < 0.001) [75]. HS symptom severity strongly correlated with negative impact on relationships [69]. A significant difference in the overall mean DLQI score was observed between presence of boils in the preceding month (mean DLQI = 13.6) and no boils (8.3, adjusted *p* = 0.001), higher overall disease-related distress score (boils score = 13.9, no boils = 6.3 adjusted *p* < 0.001), involvement of the groins (13.0, no involvement = 8.7 adjusted *p* = 0.035), number of anatomical regions involved (0–1 anatomical regions involved = 9.8, 2 regions = 12.4, ≥3 regions = 14.5, adjusted *p* = 0.007) and diabetes (diabetes present = 15.2, not present =11.5, adjusted *p* = 0.043). Disease severity (Hurley stage), younger age, diabetes, recent and increasing disease activity and specific anogenital localization were major aggravating factors [65]. Patients with involvement of the back of the neck when compared to those without nape involvement had greater disease severity, earlier disease onset, stronger family history of HS, lower body mass and higher DLQI and pain scores [76]. HRQoL impairment was associated with specific comorbidities, such as obesity and back pain, but not with having a family history of HS [77]. HS patients with alexithymia or borderline alexithymia when compared to non-alexithymic patients had significantly higher scores on the Skindex-17 psychosocial scale and the DLQI, and a lower score on the mental component of the SF-36 [78]. In HS patients with depression, disease severity as measured by the IHS4, and QoL measured by Skindex-17 and DLQI and SF-36 were significantly worse compared to HS patients with no depression [79]. When comparing the QoL impact of HS to that of psoriasis, the Skindex-17 scores of patients with HS were higher than those of psoriasis patients on 16 of the 17 Skindex items [80]. In the adjusted analysis, patients with HS who had previously not used opioids had 1.53 times the odds of using long-term opioids compared with controls (*p* < 0.001) [81]. Among patients with HS, older age and a history of ever smoking were associated with higher odds of long-term opioid use. Disciplines prescribing the most opioids to patients with HS included primary care (72.8%), anaesthesiology/pain management (8.8%), gastroenterology (4.6%), surgery (4.2%) and emergency medicine (1.8%); patients with HS may be at high risk of long-term opioid use [81]. Patients with HS had significantly lower mean Body Image Quality of Life Inventory (BI-QoL) scores than controls. Predictors of negative BI-QoL scores in patients with HS were increased body mass index, female sex and symptoms of depression [82].

## 4. Discussion

The DLQI, a dermatology-specific HRQoL instrument, remains the most widely used instrument in studies of QoL in HS. We have also identified six HS-specific QoL instruments that contain from 10 to 44 items. Amongst these, the HiSQOL and HSIA have a one-week recall period, the HSQoL-24 has a four-week recall period, the HS-QoL has a six-month recall period and the HSBOD has two parts with different recall periods: ‘the last 4 weeks′ and ‘the entire time of having HS′. The recall period for the HIDRAdisk was not stated in the original publication. HS-specific HRQoL instruments with short recall periods may better reflect treatment-related changes in clinical trials. In contrast, instruments with recall periods of six months, or covering the entire time of having HS, may be essential to study the wide range of HS effects on patients′ HRQoL and to understand the lifelong impact of HS. There has been some initial validation of HIDRAdisk, HSIA, HiSQOL and HSQoL-24, but there is still little experience of their use. Wider validation studies would be useful in the development of these instruments.

The specific validation characteristics of HRQoL instruments are important parameters in distinguishing between different measures. Well-validated instruments have a better chance of being widely used [83]. The ability to use a validated system to interpret scores and to know the MCID are other important aspects when choosing which HRQoL instrument to use. For example, the DLQI′s MCID (a score change of four points) may be used as a marker of minimal treatment efficacy [15]. This approach was used in the evaluation of medical and surgical treatments for HS using real-life data from the Scandinavian registry. HS patients treated surgically achieved the DLQI MCID two times more frequently than patients treated medically [52].

There is currently much attention being given to the concept of identifying and promoting core outcome measure sets, including measures of HRQoL, for use across all clinical studies of particular conditions [83]. In one study, the SF-36, the very widely used generic QoL instrument, did not accurately capture QoL impairment due to HS [84]. It seems probable that a combination of dermatology-specific and HS-specific instrument(s) may be recommended for inclusion into core outcome measure sets. However, sometimes, no existing HRQoL instrument may be considered to be ideal for a particular purpose. The selection process to choose measures for core outcome measurement sets should follow the highest quality methodology, including taking steps to minimise bias [85]. Ideally, instruments should only be recommended to be included in core outcome sets after they have been extensively validated.

The development and validation of different language versions of existing HRQoL instruments raises some important issues. The problem of cross-cultural inequivalence may arise [86]. International studies have demonstrated that results obtained even in neighbouring countries may not be equivalent [87]. Large international comparative studies are needed to confirm that existing instruments may be used validly in different cultural and geographical contexts. Rather than developing instruments in one country and then adapting and validating such instruments in different cultural settings, another option is to create novel QoL instruments from the start in an international context, with development being shared across different countries or cultures, as occurred in the development of the InToDermQoL [88,89] and its epidermolysis bullosa-specific module [90,91]. In line with this concept, the HS-specific HRQoL instrument HiSQOL was developed simultaneously in Denmark and the USA [32].

There is a problem of a lack of concordance between the reporting of HRQoL impact of skin diseases by children themselves and by adults on their behalf [92]. HS occurs mainly in older children and it is, therefore, usually possible to avoid proxy ratings and collect data directly from patients. Children with HS reported significant impact of HS on their lives. However, this impact may be less severe than that experienced by adult HS patients [63,64].

The interpretation of data on gender differences in HS raises issues that are difficult to resolve, as in many other chronic skin diseases. However, it seems that female patients tend to experience a greater HRQoL impact than males. To confirm this and to remove confounding factors, it would be necessary to undertake large population studies or at least studies where participants are well matched by factors other than gender [93,94].

In the publications from 2019–2021 reviewed in this study, data were presented on many different aspects of the HRQoL impact of HS, including the impact on family members and the improvement in HRQoL that may result from effective treatment. Four studies from different countries reported a significant impact of HS on patients′ partners and family members [60,61,62,63]. The Family Dermatology Life Quality Index (FDLQI) scores in those studies ranged from 8.7 to 13.8 (FDLQI possible score range = 0 [no impact] to 30 [maximum impact]). Several studies have demonstrated a positive correlation between DLQI scores and HS severity (Hurley stages and modified Hurley stages) [65,69,70,71]. Many HS patients in these studies were unemployed and of those who had a job, many reported problems with presenteeism and overall work impairment that increased along with HS severity [64,65,66,67,68,69]. Several recent studies have shown significant HRQoL improvement in patients treated with adalimumab [40,41,42,43,45,46]. Smaller studies on ustekinumab [48] and secukinumab [49] also showed a positive effect on patients′ HRQoL. A placebo-controlled trial found that apremilast had no greater impact on HRQoL than placebo [38]. However, an open-label study showed significant improvement of DLQI scores with apremilast [39]. Several other treatment methods also demonstrated a positive effect on patients′ HRQoL, but it seems that surgery still has the greatest long-term benefit on HRQoL in HS patients [52].

There are many reasons to use HRQoL assessment in clinical practice. Members of the EADV Task Force on Quality of Life independently listed the ways they thought that it may be advantageous to measure QoL in routine dermatological clinical practice: a total of 108 different ways of using QoL information in clinical practice were suggested [17]. The majority of these suggested advantages of measuring QoL are applicable to the routine clinical management of HS. Both patients and dermatologists prefer to use instruments with only a few questions, and it is crucial that only a short time is needed for their completion.

## 5. Conclusions

HS has a profound impact on the lives of patients and their family members. Several factors, such as the presence of comorbidities, unemployment and HS severity, may contribute to making this impact even more severe. The instrument most widely used to measure this impact is the DLQI, a dermatology-specific measure. We also identified six HS-specific HRQoL instruments. Of them, HIDRAdisk, HSIA, HiSQOL and HSQoL-24 are the better-validated instruments, but there is still a lack of experience of their use. Several treatment methods result in a positive effect on patients′ HRQoL. Surgery remains the method that provides the greatest benefit to HRQoL. Several studies have been published over the last three years that confirm a positive effect of adalimumab on the HRQoL of patients with HS. There are data on the influence of several other biologics on the HRQoL of HS patients, but these studies are mostly small or have not been confirmed.

## Figures and Tables

**Table 1:** HS-specific HRQoL instruments.

Name	Short Name/ Abbreviation	Number of Items	Number of Scales	Recall Period	Scoring	Validation
Quality of Life for Hidradenitis Suppurativa [30]	HS-QoL	44	7 subscales: ‘physical consequences’, ‘HS symptoms’, ‘sexual activity consequences’, ‘emotional consequences’, ‘social consequences’, ‘work consequences’ and ‘social support’	Last 6 months	Five-point scale ranging from 1 (never) to 5 (all the time)	Internal consistency and convergent validity
Hidradenitis Suppurativa Burden of Disease [31]	HSBOD	19	5 domains: ‘symptoms and feelings’, ‘daily activities’, ‘leisure’, ‘work/school’ and ‘personal relationships’	Two parts with different recall periods: the last 4 weeks (14 items) and the entire time of having HS (5 items)	Visual analogue scale from 0 to 10	Internal consistency and convergent validity
Hidradenitis Suppurativa Quality of life [32]	HiSQOL	17	3 subscales: ‘symptoms’, ‘activity-adaptation’ and ‘psychosocial’	Last week	Five-point scale ranging from 0 (not at all) to 4 (extremely)	Internal consistency, convergent validity, and test-retest reliability
HSQoL-24 [33]	HSQoL-24	24	6 domains: ‘psychosocial’, ‘economic’, ‘occupational’, ‘relationships’, ‘personal’ and ‘clinical’	Last 4 weeks	Five-point scale ranging from 0 (never) to 4 (always)	Internal consistency, convergent validity, and sensitive to change
HIDRAdisk [34]	HIDRAdisk	10	Unidimensional	Not stated	Visual analogue scale from 0 to 10	Internal consistency, convergent validity, test-retest reliability, and sensitive to change
Hidradenitis Suppurativa Impact Assessment [35]	HSIA	18	Unidimensional	Last week	Items 1–16 are scored on a 0 (no impact) to 10 (extreme impact) scale. Items 17 and 18 are not included in the HSIA total score.	Internal consistency, convergent validity, and test-retest reliability

## Data Availability

The data that support the findings of this study are available from the corresponding author, upon reasonable request.
